# Detailed Profiling of the Tumor Microenvironment in Ethnic Breast Cancer, Using Tissue Microarrays and Multiplex Immunofluorescence

**DOI:** 10.3390/ijms25126501

**Published:** 2024-06-13

**Authors:** Mohamed Zaakouk, Aisling Longworth, Kelly Hunter, Suhaib Jiman, Daniel Kearns, Mervat El-Deftar, Abeer M Shaaban

**Affiliations:** 1 Institute of Cancer and Genomic Sciences, University of Birmingham, Birmingham B15 2TT, UK; m.zaakouk@bham.ac.uk (M.Z.); kelly.hunter@propath.co.uk (K.H.); 2 Department of Cellular Pathology, Queen Elizabeth Hospital Birmingham, Birmingham B15 2GW, UK; aisling.longworth@nhs.net (A.L.); suhaibasif.jiman@uhb.nhs.uk (S.J.); daniel.kearns@uhb.nhs.uk (D.K.); 3 Cancer Pathology Department, National Cancer Institute, Cairo University, Cairo 11796, Egypt; mohamed.zaakouk@nci.cu.edu.eg

**Keywords:** tumor microenvironment, multiplex immunofluorescence, ethnic breast cancer, multispectral imaging, TILs, T-helper cells, Treg cells, FOXP3, TAMs, B cells

## Abstract

Breast cancer poses a global health challenge, yet the influence of ethnicity on the tumor microenvironment (TME) remains understudied. In this investigation, we examined immune cell infiltration in 230 breast cancer samples, emphasizing diverse ethnic populations. Leveraging tissue microarrays (TMAs) and core samples, we applied multiplex immunofluorescence (mIF) to dissect immune cell subtypes across TME regions. Our analysis revealed distinct immune cell distribution patterns, particularly enriched in aggressive molecular subtypes triple-negative and HER2-positive tumors. We observed significant correlations between immune cell abundance and key clinicopathological parameters, including tumor size, lymph node involvement, and patient overall survival. Notably, immune cell location within different TME regions showed varying correlations with clinicopathologic parameters. Additionally, ethnicities exhibited diverse distributions of cells, with certain ethnicities showing higher abundance compared to others. In TMA samples, patients of Chinese and Caribbean origin displayed significantly lower numbers of B cells, TAMs, and FOXP3-positive cells. These findings highlight the intricate interplay between immune cells and breast cancer progression, with implications for personalized treatment strategies. Moving forward, integrating advanced imaging techniques, and exploring immune cell heterogeneity in diverse ethnic cohorts can uncover novel immune signatures and guide tailored immunotherapeutic interventions, ultimately improving breast cancer management.

## 1. Introduction

Studying the immune tumor microenvironment (TME) of breast cancer among patients of different ethnicities is a crucial area of research, especially considering the potential impact of ethnic diversity on disease progression, treatment response, and clinical outcomes.

Patients of non-Caucasian origin are significantly underrepresented in research and trials of breast cancer, making the profiling of breast cancer in non-Caucasian women an urgent unmet need. Differences in the presentation and outcome of women with breast cancer of different backgrounds were recently highlighted. For example, black women presenting with node negative breast cancer were more likely to die of the disease compared with non-Hispanic Whites with the same 21 Gene Recurrence Score [1,2]. Additionally, disparities in breast cancer incidence, stage at diagnosis, and treatment outcomes have been reported across ethnicities [3,4]. Understanding how the immune TME varies among different ethnic groups can therefore provide valuable insights into the underlying biological mechanisms driving breast cancer disparities.

Few studies have highlighted the significance of ethnicity in shaping the immune landscape of breast tumors. For example, variations in the expression of immune-related genes, the distribution of tumor-infiltrating lymphocytes (TILs), and the immune checkpoint expressions among different ethnic groups have been reported [5,6]. Understanding the immune TME in ethnically diverse breast cancer populations is essential for developing personalized treatment strategies and reducing disparities in cancer care. By elucidating the immune mechanisms underlying ethnic disparities in breast cancer, researchers can identify novel biomarkers, therapeutic targets, and intervention strategies tailored to specific ethnic groups [7,8,9]. While the overall immune infiltrate can be assessed by examination of hematoxylin and eosin (H&E)-stained tumor section, as per the International TILs Working Group criteria [10], this does not allow for the phenotyping of the various cells included, nor studying marker co-expression.

Ethnicity-related differences in the immune TME may stem from various factors, including genetic variations, socio-economic determinants, access to healthcare, lifestyle factors, and environmental exposures. These differences could influence immune cell infiltration, cytokine profiles, tumor-immune interactions, and ultimately, disease progression and response to therapy, particularly neoadjuvant chemotherapy and immunotherapy [11].

Advances in immunotherapy have revolutionized the treatment landscape for various cancers, including breast cancer. However, the response to immunotherapy can vary significantly among individuals, influenced by factors such as tumor biology, immune landscape, and patient-specific characteristics, including ethnicity. Recent studies have underscored the importance of immune checkpoint inhibitors, such as PD-1/PD-L1 and CTLA-4, in unleashing the anti-tumor potential of the immune system [12,13,14]. However, the efficacy of these therapies in ethnically diverse populations remains underexplored. It is crucial to investigate whether variations in the immune TME across different ethnicities affect the response to immunotherapy. For instance, certain genetic polymorphisms and immune gene expression profiles that differ among ethnic groups might influence the effectiveness of immune checkpoint blockade [13,15]. Additionally, understanding the role of the microbiome, which can vary significantly with ethnicity, in modulating the immune response and influencing immunotherapy outcomes is an emerging area of interest [16,17]. Addressing these gaps in knowledge can lead to more effective and equitable cancer treatment strategies, ensuring that advancements in cancer therapy benefit all populations.

The tumor microenvironment (TME) is a complex milieu composed of cancer cells, stromal cells, extracellular matrix components, and immune cells. Among these constituents, immune cells play a pivotal role in shaping the tumor’s fate, with implications for disease progression, metastasis, and therapeutic response. Of the TME components, T-helper cells play a critical role in the complex tumor microenvironment (TME) of breast cancer, influencing both antitumor immunity and immunosuppressive processes. Their diverse subtypes, such as Th1, Th2, and regulatory T cells (Tregs), contribute to the dynamic immune landscape. T-helper cells play a crucial role in the complex tumor microenvironment (TME) of breast cancer, influencing both antitumor immunity and immunosuppressive processes. Their diverse subtypes, such as Th1, Th2, and regulatory T cells (Tregs), contribute to the dynamic immune landscape.

T cytotoxic cells are effector cells that play a critical role in recognizing and eliminating tumor cells through the release of cytotoxic molecules and the induction of apoptosis. B cells, on the other hand, can modulate the TME through interactions with other immune cells, cytokine secretion, and antigen presentation. Tumor-associated macrophages (TAMs) exert diverse functions that profoundly influence tumor progression and metastasis. TAMs, predominantly of the M2-like phenotype, promote tumor growth, angiogenesis, and immunosuppression while inhibiting antitumor immune responses. Forkhead box P3 (FOXP3) is a crucial transcription factor primarily associated with regulatory T cells (Tregs), and also plays a significant role in breast cancer beyond its involvement in Treg cells. Emerging evidence suggests that FOXP3 expression is not limited to Tregs but is also observed in various cancer cells, including breast cancer, where aberrant FOXP3 expression has been implicated in promoting tumor growth, metastasis, and immune evasion mechanisms [18,19,20,21,22].

Recent advancements in molecular and imaging technologies have enabled a more detailed analysis of the TME, providing insights into the spatial organization and functional states of immune cells within tumors. High-dimensional techniques, such as single-cell RNA sequencing and multiplex immunofluorescence, allow for the comprehensive profiling of the immune landscape at single-cell resolution. These approaches have revealed substantial heterogeneity in immune cell populations and their interactions within the TME, further highlighting the importance of considering ethnic diversity in breast cancer research [23,24,25,26].

Several lines of evidence suggest that breast tumors are subject to immune surveillance mechanisms, whereby infiltrating immune cells can recognize and eliminate malignant cells. However, the dynamic interaction between cancer cells and the immune system often results in immune evasion and tumor immune privilege, fostering tumor growth and dissemination [27,28,29,30]. We therefore sought to analyze in detail the microenvironment, including T-helper, T regulatory, T cytotoxic cells, B lymphocytes and Tumor Associated Macrophages (TAM), within a large, well-characterized cohort of women of non-Caucasian origin. In addition to the composition, we analyzed the topography and colocalization of the immune cells within the tumor microenvironment.

## 2. Results

### 2.1. Cohort Characteristics

Cohort clinical and pathological characteristics of the two sets of samples (core (*n* = 87) and TMA (*n* = 143)) and per patient (*n* = 192) are shown in Table 1. Most tumors (89.6%) were non-low-grade with a significant proportion of HER2-positive and triple-negative phenotype (17.7% each). More than half (55.3%) of the carcinomas were of T1 stage; 37% of the tumors had associated metastatic axillary lymph nodes. Follow-up ranged from 7 to 275 months, with a median of 103 months (IQR 86.25:124.75). The five- and ten-year survival rates were 88% and 78%, respectively.

**Figure 1:**
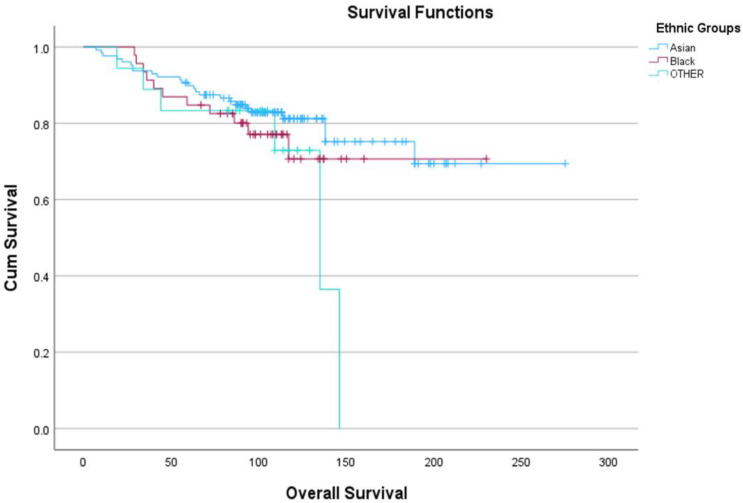
Kaplan–Meyer survival curves representing OS of the three ethnic groups, Asian, Black and OTHER.

The 192 patients belonged to different ethnicities and were distributed in descending order as follows: Pakistani (66; 34.4%), Indian (42; 21.9%), Caribbean (24; 12.5%), African (14; 7.3%), Chinese (6; 3.1%) and Bangladeshi (1; 0.5%) (Table 2). These ethnicities were also grouped into three broader categories, Asian, Black and OTHER, for statistical analysis. The five-year OS ranged from 83.5 to 90.5% and 10-year OS ranged from 71 to 81% (Figure 1).

### 2.2. Dominant Immune Cell Phenotypes in Different Regions of the Stained Cases

In the core samples, T-helper cells were the dominant immune cell phenotype in 71.3% (62/87), while serving as the second predominant phenotype in 19.5% of samples (17/87). Cytotoxic T cells were dominant in 14.9% (13/87) and the second most predominant in 46% (40/87). Both B-cells and TAMs were each the dominant phenotype in 5.6% of cases, while being the second most predominant in 13% (Table 3).

In the TMA samples, T-helper cells were also the dominant immune cell phenotype in 69.2% (99/143), and the second most predominant in 18.9% of samples (27/143). Following this, B-cells were dominant in 18.9% (27/143) and the second most predominant in 10.5% (15/143). Cytotoxic T cells emerged as the second most predominant phenotype in 60.8% (87/143), while being the dominant phenotype in 6.3% of cases. TAMs were dominant in 5.6% and the second most predominant in 9.8% of cases.

### 2.3. Distribution of Immune Cells in Relation to the Histopathologic Characteristics

The distribution of different immune cells’ medians of counts, percentages, and densities in each region (stromal, tumoral and total) was evaluated across the different histopathologic characteristics.

#### 2.3.1. Core Samples (Table S1 and Figure 2a)

T-helper cells (Th) exhibited significantly higher counts and density in HER2-positive tumors compared to TNBC and Luminal tumors, particularly in stromal and tumoral regions. Moreover, a pronounced density of T-helper cells was observed in stromal and total regions of grade 3 tumors. Stromal counts differed significantly across different ethnicities. Regulatory T cells (Treg) showed increased counts in grade 3 tumors across tumoral and total regions, with elevated density in stromal and total regions of grade 3 tumors and tumors that did not receive neoadjuvant chemotherapy (NACT). Cytotoxic T cells (Tc) were more abundant in TNBC tumors, compared to Luminal and HER2-positive tumors across stromal, tumoral, and total regions. B cells displayed higher counts and densities in TNBC tumors and grade 3 tumors across all regions. Stromal and total counts differ among different ethnicities.

Tumor-associated macrophages (TAMs) counts were more prevalent in TNBC subtype (stromal and total) and grade 3 tumors (tumoral). Tumoral TAMs were more prevalent in cases that did not receive NACT. Tumoral density differed significantly among the ethnic groupings, being higher in Asian than in Black and OTHER groups. FOXP3-positive cells were enriched in grade 3 tumors and Luminal subtype tumors in tumoral and stromal regions, respectively. Total percentage and density were higher in cases who did not receive NACT. Immune cell populations (IMM) showed varying counts among different ethnicities, with higher total percentages in HER2-positive tumors and increased stromal density in both TNBC and grade 3 tumors. Total cell population (ALL) demonstrated variations among ethnicities with higher counts and density in TNBC and grade 3 tumors. Details and *p* values are shown in Table S1.

#### 2.3.2. TMA Samples (Table S2 and Figure 2b)

T-helper cells (Th) counts in each of the studied regions were higher in grade 3 tumors and TNBC tumors compared to Luminal and HER2 positive types. The percentage of Th cells declined with decreasing tumor grade. Similarly, Th density was higher in grade 3 tumors and TNBC. Regulatory T cells (Treg) counts were notably elevated in TNBC relative to Luminal and HER2-positive types across all regions, and were higher in grade 3 tumors compared to grades 1 and 2. The percentage of Treg cells also decreased with decreasing tumor grade. The density of Treg cells showed a similar pattern, being higher in grade 3 tumors, and more pronounced in TNBC compared to other types. Cytotoxic T cells (Tc) showed variability among different ethnicities, with counts decreasing as tumor grade decreased. The percentage and density of Tc cells were higher in grade 3.

B cells counts varied significantly according to the T-stage and ethnicities, with distinct percentages observed among different ethnic groups. The density of B cells showed variations among ethnicities and nodal stages, with differential patterns observed in stromal and tumoral regions. Tumor-associated macrophages (TAMs) counts were notably higher in grade 3 tumors compared to grades 1 and 2 and were elevated in TNBC compared to Luminal and HER2-positive types. The percentage of TAMs exhibited a gradual decrease from grade 3 to grade 1, with variations among ethnicities. TAM density showed similar trends, being higher in grade 3 tumors compared to grades 1 and 2, and varying among molecular types and nodal status. Total percentages differed significantly among the ethnic groupings, being the highest in OTHER and lowest in Black. FOXP3-positive cells counts displayed variations among ethnicities, indicating differential distributions across different ethnic groups.

Immune cell populations (IMM) counts were higher in grade 3 tumors compared to grades 2 and 1 across stromal, tumoral, and total regions, suggesting a higher immune cell infiltration in more aggressive tumors. The percentage of IMM cells exhibited a decreasing pattern from grade 3 to grade 1 in stromal and tumoral regions. IMM density was higher in tumors with grade 3 compared to grades 2 and 1 across stromal, tumoral, and total regions. Total cell population (ALL) counts were elevated in grade 3 tumors and those who did not receive NACT, with grade 3 compared to grades 2 and 1 across stromal, tumoral, and total regions, indicating a higher overall immune cell presence in more aggressive tumors. The density showed contrasting patterns according to the location of cells, where stromal density was higher in TNBC and grade 3 tumors, while tumoral density was higher in Luminal and grade 1 tumors. Details and *p* values are shown in Table S2.

### 2.4. Statistical Correlation between TME Immune Cells, Histopathologucal Paramaters and Patient Outcome

The relations between different immune cells counts, percentages and densities in each region (stromal, tumoral and total) and the histopathologic characteristics and overall survival (OS) were examined.

#### 2.4.1. Core Samples (Table S3 and Figure 3)

The density of T-helper cells in the tumoral region exhibited a significant positive correlation with OS, indicating association with improved OS. B cells demonstrated significant positive associations with OS. The count, percentage, and density of B cells in the tumoral region exhibited positive correlations with OS. Additionally, the density of B cells in the stromal region significantly correlated positively with OS. The density of immune cells (IMM) in the tumoral region showed a significant positive correlation with OS, suggesting a potential association with improved OS. The density of total cell population (ALL) in the stromal and total regions demonstrated significant positive correlations with OS. Furthermore, the density of all cells in the tumoral region showed a notable positive correlation with age at diagnosis. Details and *p* values are shown in Table S3.

#### 2.4.2. TMA Samples (Table S4 and Figure 4)

T-helper cells: Positive associations were observed between the counts, percentage, and density of T-helper cells in both stromal and total regions and OS, indicating a potential prognostic value for higher T-helper cell presence. B cells exhibited negative correlations with lymph node (LN) count and tumor size, but a positive association with age at diagnosis in the stromal region. Similar negative correlations with LN count and tumor size were observed in the total region for counts, percentage, and density of B cells. Higher B cell density in the stromal region was associated with older age at diagnosis. TAMs counts in stromal and total regions negatively correlated with LN count and tumor size. Tumoral TAMs percentage exhibited a negative correlation with OS, while TAMs density correlated negatively with LN count and tumor size, yet positively with OS.

Immune cells (IMM) counts in the total region negatively correlated with tumor size. The percentage of stromal immune cells correlated negatively with tumor size but positively with OS. The density of immune cells in both stromal and total regions showed a negative correlation with tumor size. A higher density of total cells (ALL) in the total region was associated with smaller tumor size and better OS, suggesting a potentially favorable prognosis with increased cell density. Details and *p* values are shown in Table S4.

### 2.5. Summary of Findings (Figure S1)

With the exception of FOXP3-positive cells, all immune cell phenotypes showed varying distributions among different molecular subtypes, with the highest counts observed in the triple-negative breast cancer (TNBC) subtype. Similarly, regarding tumor grades, all phenotypes exhibited statistically significant differences in distribution, with the highest expression identified in grade 3 tumors. Conversely, T-stage, N-stage, and nodal status did not appear to significantly affect the distribution of immune cells. Interestingly, B cells and tumor-associated macrophages (TAMs) correlated with a lower number of involved lymph nodes and smaller tumor size. The latter also correlated negatively with the overall sum of studied immune cells (IMM) and the total number of cells (ALL).

### 2.6. Ethnic Groupings and Individual Ethnicities (Tables S1–S4 and Figure 2 and Figure S2)

When categorizing ethnicities into three broad categories (Asian, Black, and OTHER groups), significant differences were observed only in TAMs. In core samples, TAMs tumoral density varied significantly (*p* < 0.04) among the ethnic groups, with higher densities observed in Asian individuals compared to those of Black and OTHER ethnicities. Similarly, in TMA samples, the total percentage of TAMs differed significantly (*p* < 0.002) among the ethnic groups, with the highest percentage found in individuals categorized as OTHER and the lowest in those classified as Black.

The detailed analysis of the distribution of immune cell phenotypes among individual ethnicities revealed additional statistically significant disparities across all studied immune cells except for regulatory T cells (Tregs). In core samples, patients of Chinese and Caribbean descent exhibited higher numbers of Th and B cells, compared to individuals of other ethnicities within the Asian and Black groupings, respectively. Moreover, patients of Chinese descent also showed elevated levels of IMM and ALL cells. Conversely, in TMA samples, patients of Chinese and Caribbean origin displayed lower numbers of B cells, TAMs, and FOXP3-positive cells.

## 3. Discussion

In the current study, we analyzed in detail the tumor microenvironment phenotype and colocalization in a large cohort of non-Caucasian breast cancer. We used a state-of-the-art multiplex immunofluorescence technology and artificial intelligence (AI) software algorithms for the simultaneous quantitative analysis of relevant immune cells, and showed differential expression based on tumor characteristics and significant correlation with overall survival and patient’s outcome. Several studies found a strong linear relationship between an increase in tumor-infiltrating lymphocytes (TILs) and improved recurrence-free survival for triple-negative and HER2-positive breast cancers [31,32]. Higher levels of TILs correlated in all molecular subtypes with increased rates of pathologic complete response to neoadjuvant therapy [32,33].

### 3.1. Th Cells

T-helper (Th) cells play a pivotal role in orchestrating the immune response against tumors, exhibiting both antitumor and protumor functions. Th1 cells, known for promoting antitumor immunity by activating cytotoxic T lymphocytes (CTLs) and enhancing macrophage-mediated tumor cell killing [34,35], can paradoxically contribute to tumor progression by fostering an immunosuppressive microenvironment, thereby aiding cancer cells in evading immune surveillance [36,37,38,39,40,41,42,43]. Our study delved into the intricate role of Th cells in breast cancer pathogenesis, revealing their differential abundance across molecular subtypes and tumor grades. Notably, Th cells were more prevalent in aggressive subtypes such as triple-negative breast cancer (TNBC) and HER2-positive tumors, particularly in high-grade lesions.

However, the higher infiltration of Th cells was associated with improved overall survival (OS), indicative of their complex involvement in tumor immunity. Our findings corroborate previous studies demonstrating that the increased infiltration of both CD4 and CD8 cells correlated with favorable disease outcomes, as these cells synergistically mediate robust antitumor immune responses [37,44]. Others found that a higher infiltration by CD4 cells was associated with increased nodal involvement [45,46,47]. This could be attributed to the increased vasculogenesis and release of immunosuppressive cytokines [47]. Similar findings of associated worse prognosis with CD4 cells have been reported in other cancers, such as glioblastomas [48]. These insights underscore the dual nature of Th cells in breast cancer immunobiology, and highlight their significance as both prognostic indicators and therapeutic targets.

### 3.2. Treg Cells

Regulatory T cells (Tregs) play a crucial role in suppressing the immune system, thereby preventing excessive immune responses that could lead to collateral damage to normal tissues. Their inhibitory function extends to effector T cells, limiting their activation and proliferation, which in turn curtails their ability to effectively target and eliminate tumor cells. Numerous studies have highlighted the accumulation of Tregs in breast cancer tissues, correlating with tumor progression, metastasis, and poorer patient outcomes [22,49].

In the current study, we observed the higher infiltration of T-regulatory cells, indicated by increased counts, percentages, and density across all regions, exhibiting variations among molecular subtypes and tumor grades, particularly favoring triple-negative breast cancer (TNBC) and grade 3 tumors. Consistent with previous findings, elevated Treg cell counts and density have been associated with larger tumor size, higher tumor grade, and shorter overall survival [49]. However, this is contrary to other studies that found that high densities of Tregs, Tc and B cells correlate with better OS [36,50]; the former study was conducted in Singapore.

Additionally, Asano et al., 2016 [51], reported lower rates of pathological complete response (pCR) in breast cancer patients with higher Treg cell infiltration. However, it is speculated that neoadjuvant chemotherapy may suppress FOXP3 expression, thereby alleviating immune suppression and potentially leading to a better response to CTH and an increased likelihood of achieving pCR [52,53]. In some contexts, the depletion of Treg cells has been associated with enhanced anti-tumor immune responses and improved outcomes in cancer patients [54,55].

### 3.3. Tc Cells

In breast cancer, the infiltration of T cytotoxic cells into the tumor microenvironment (TME) has been linked to improved patient prognosis and enhanced antitumor immune responses. These CD8 cytotoxic T lymphocytes serve as the primary agents for eliminating cancer cells by recognizing specific epitopes presented on major histocompatibility complex (MHC) class I molecules. Upon recognition, CD8+ T cells release perforin and granzymes, triggering cancer cell death.

Our current work revealed variations in CD8-positive cytotoxic T cells among different molecular subtypes and grades, with higher counts, percentages and densities observed in the more aggressive types, particularly triple-negative breast cancer (TNBC) and HER2-positive cancers, as well as grade 3 tumors. Similarly, Tsiatas et al. [56] found that CD8 cells were more prevalent in higher-grade tumors of the HER2-positive and TNBC subtypes [57], which paradoxically exhibited a better response to chemotherapy CTH [58]. Moreover, others reported that CD8 cells exert anti-tumor effects in low-grade cancers [51,59,60,61].

In various cancers, including melanoma, colorectal cancer (CRC), and non-small-cell lung cancer (NSCLC), the presence of CD8 cells has been associated with a favorable response to immune checkpoint inhibitors [62,63,64,65]. However, the prognostic role of CD8 cells in breast cancer is complex, while their presence may signify immune activation leading to better disease-free survival (DFS) DFS [66]. Some studies, including that by Wang et al. in 2018 [67], suggested that high levels of CD8 cells may indicate an exhausted T cell phenotype, leading to T cell dysfunction and a compromised immune response, resulting in worse DFS. This dysfunction has been linked to elevated levels of proteins such as LAG-3 and PD-1.

Furthermore, the molecular subtype of breast cancer can influence the impact of CD8 cells on disease recurrence and patient outcomes, and this is particularly relevant in estrogen receptor-positive (ER+) cancers [33,68,69]. Overall, the intricate interplay between CD8 cytotoxic T cells and the tumor microenvironment highlights the complexity of immune responses in breast cancer, and underscores the need for personalized therapeutic approaches.

### 3.4. B Cells

While some studies proposed that B cells play a role in antitumor immunity by facilitating immune surveillance and mediating the antibody-mediated destruction of tumor cells, others suggested a pro-tumorigenic function characterized by immunosuppressive activities and the support of tumor growth. The interplay between B cells and the tumor microenvironment (TME) highlights their potential use as both prognostic markers and therapeutic targets in breast cancer [70,71,72]. B cells are prevalent in nearly a quarter of breast cancers and constitute up to 40% of the total tumor-infiltrating lymphocytes (TILs) population. Apart from their classical role in antibody production, B cells express MHC class II proteins, enabling them to present antigens to CD4+ T cells and modulate T cell-mediated anti-tumor activity [73,74].

Our analysis revealed higher counts, percentages and densities in each of the studied regions in TNBC and grade 3 cancers. Although they were more prevalent in the more aggressive tumors, stromal and total B cells correlated with smaller tumor size and a lower number of involved lymph nodes. In relation to OS, stromal density and tumoral counts were associated with better OS, compared to tumoral percentage and density, which correlated with shorter OS.

This aligns with other studies, which found that CD20 expression levels were identified in T1 stage tumors, but were also detected in higher-grade cancers, particularly in HER2-positive and TNBC subtypes [75]. Notably, these cells were associated with a favorable prognosis and a predictive value, often linked to a higher percentage of pathological complete response (pCR) [73,76,77,78].

In other cancers such as colorectal cancer and non-small-cell lung cancer (NSCLC), the presence of CD20 cells has been associated with a favorable prognosis [79,80]. Moreover, the co-presence of many CD4 and CD8 cells, as well as many CD20 and CD8 cells, has been correlated with better disease-free survival (DFS) and overall survival (OS) [36,50].

### 3.5. TAMs

The presence of tumor-associated macrophages (TAMs) within breast tumors is strongly linked to poor clinical outcomes and resistance to therapy. TAMs play a crucial role in establishing an immunosuppressive tumor microenvironment (TME) by secreting cytokines, growth factors, and enzymes involved in extracellular matrix remodeling [81,82]. Breast cancer cells actively recruit and activate CD68+ TAMs by secreting immunosuppressive cytokines, thereby fostering an immune environment conducive to tumor growth and dissemination [83,84,85]. These macrophages contribute to immune evasion by producing cytokines that inhibit the infiltration of cytotoxic T cells into the tumor region [84,86,87].

Our study revealed abundant counts and density in grade 3 and TNBC tumors. However, they correlated negatively with tumor size and the number of involved lymph nodes. Similar to different associations, tumoral density correlated with better OS, compared to total percentage. Our results align with previous studies of Caucasian breast cancer that have found that TAMs are associated with poor prognostic factors and survival. Medrek et al. found that a higher stromal density of TAMs is associated with increased tumor size and higher-grade tumors, as well as shortened overall survival [88]. Gwak et al. found that TAMs correlate with aggressive outcome, irrespective of their location within the tumor [89]. Others found that higher TAMs correlated with shortened overall survival as well as disease-free survival and relapse-free survival, respectively [77,90]. Shorter DFS and OS could be attributed to the inflammatory cytokines and chemokines produced by TAMs, which contribute to tumoral growth and metastasis [91,92].

### 3.6. FOXP3-Positive Cells

FOXP3 is a crucial transcription factor primarily associated with regulatory T cells (Tregs), and also plays a significant role in breast cancer beyond its involvement in Treg cells. Emerging evidence suggests that FOXP3 expression is not limited to Tregs but is also observed in various cancer cells, including breast cancer, where aberrant FOXP3 expression has been implicated in promoting tumor growth, metastasis, and immune evasion mechanisms [20,21,22,93]. Moreover, FOXP3-positive breast cancer cells have been associated with poorer clinical outcomes and resistance to therapy. Therefore, understanding the multifaceted roles of FOXP3 in breast cancer pathogenesis may offer insights into novel therapeutic strategies targeting this transcription factor, aiming at suppressing tumor progression and improving patient outcomes in breast cancer [21,94].

Our study found that a higher stromal percentage was found in luminal cancers, compared with HER2-positive and TNBC tumors. The tumoral count was higher in grade 3 tumors. However, higher FOXP3 stromal count and percentage were associated with shortened OS, while higher density correlated with improved OS. Previously, higher FOXP3 expression has been associated with larger tumor size, indicating a potential role in tumor growth and progression [95]. Additionally, increased FOXP3 levels have been correlated with greater lymph node involvement, suggesting a potential role in promoting metastasis [96]. Importantly, patients with elevated FOXP3 expression have been shown to experience poorer overall survival and disease-free survival rates compared to those with lower FOXP3 levels in non-ethnic cohorts [97]. Conversely, other studies have reported contradictory findings, linking higher FOXP3 expression with smaller tumor size, fewer lymph node metastases, and improved survival outcomes [21,93,98]. These conflicting results underscore the complexity of FOXP3′s role in breast cancer and highlight the need for further research to clarify its clinical implications.

### 3.7. Significant Findings

The cellular distribution and topography of the immune cells significantly varied among the molecular subtypes. Stromal counts, stromal density and total density of Th cells were more abundant in HER2-positive tumors compared to Luminal and TNBC tumors. On the contrary, tumoral counts and density were more abundant in the TNBC subtype. Regarding Treg cells, only stromal and total percentage were more pronounced in the HER2-positive tumors, while rest of the parameters were more abundant in the TNBC subtype. FOXP3-positive cells, which are known to correlate with worse prognosis and prevalence in the more aggressive molecular types, were found to be more abundant in the Luminal subtype, represented by stromal percentage. Moreover, the stromal density of FOXP3-positive cells differed from their count and percentage, where higher density correlated with better OS compared to higher count and percentage. This implies that immune cells could be low in count yet impact survival and prognosis based on their localization and density in the TME. The total percentage of IMM cells was higher in HER2-positive tumors, while the rest of the parameters were higher in the TNBC subtype. Similarly, the tumoral densities of ALL cells were higher in grade 1 and Luminal tumors.

Regarding racial disparities among different ethnicities, our results are concordant with other studies [99,100,101,102,103,104], which found significant differential distributions of immune cells between different ethnicities. Comparing individual ethnicities rather than grouping into three groups (Asian, Black and OTHER) showed a larger number of significant differences. These data highlight the importance of studying racial disparities in detail, rather than considering common groupings, like Black and Asian. Many of the published works in the literature studying racial disparities at the microenvironment level compare white Caucasian to Black ethnic groups. Further comparative studies inclusive of more ethnicities within a single study are needed to ensure uniform methodology and analyses of data.

### 3.8. Strengths of the Study

Studying a diverse cohort representing ethnic breast cancer populations is an important strength, as such groups are often underrepresented in the existing literature. Our work has analyzed in detail the immune microenvironment of breast cancer in different ethnicities, and the results are discussed in light of the published data on the White Caucasian population. This provides insights into potential ethnic disparities in immune cell infiltration and response to treatment, thereby contributing to more personalized and effective therapeutic strategies. The utilization of both tissue microarrays (TMAs) and individual cores allows for a comprehensive analysis of the tumor microenvironment (TME), capturing spatial heterogeneity and enabling detailed profiling in every region of interest. This approach enhances the robustness of our findings and provides a more nuanced understanding of the immune landscape within such tumors. Additionally, the extended follow-up period offers valuable longitudinal data, facilitating the assessment of long-term treatment outcomes and disease progression. Taken together, these methodological strengths have helped advance our understanding of immune-mediated mechanisms in breast cancer from patients with minority ethnic backgrounds.

### 3.9. Future Plans

Moving forward, our research aims to explore in detail the complexity of immune cell subtypes within the breast cancer microenvironment. We plan to expand our analysis by employing a wider panel of multiplex immunofluorescence (mIF) markers, allowing for the more refined subtyping of immune cell populations. Furthermore, ongoing efforts are focused on investigating the co-localization patterns of the studied immune cells, which promises to provide valuable insights into their spatial distribution and functional relationships within the TME. This comprehensive approach will enable us to elucidate intricate interactions between different immune cell subsets and their roles in tumor progression and treatment response.

## 4. Materials and Methods

### 4.1. Selection of Cases

A total of 230 samples of invasive breast cancer were obtained from patients of minority ethnic backgrounds, diagnosed at the Queen Elizabeth Hospital Birmingham, a large UK institution, between 2000 and 2016, as previously described [105]. These samples consisted of 87 core biopsies and 143 excision specimens. The initial database search of all patients, from all ethnicities, identified 7554 patients. Patients of British/White ancestry, unconfirmed ethnicity or missing clinical data (*n* = 6805) were excluded. Cases with a diagnosis of in situ carcinoma only and those without available paraffin blocks were also excluded. Excision paraffin blocks from all 143 available tumors were used to create tissue microarrays (TMAs), while paraffin blocks from 87 core biopsy cases were also processed. The selection of cases aimed to achieve a minimum 7-year follow-up period. These samples represented 192 patients, with 38 patients having both core and excision specimens. Clinical data, tumor histopathological characteristics, and patient outcomes were retrieved from electronic clinical records.

### 4.2. Tissue Microarray (TMA) Construction

A tissue microarray (TMA) comprising 143 breast carcinomas was created, organized into four blocks, in duplicates, with each tumor represented by four cores—two from the central region and two from the periphery. Cores were taken from marked areas of the donor blocks using a manual MTA (Manual Tissue Arrayer), Beecher Instruments (Sun Prairie, WI, USA). A 0.6 mm-diameter needle (with a core radius of 0.3 mm, resulting in a core area of 0.283 mm^2^) was used for punching the cores. These cores were then inserted into a new paraffin block (recipient block) with a spacing of 1.25 mm between cores at predefined array coordinates. Consequently, four tissue cores (totaling 1.132 mm^2^) were sampled from each case’s donor blocks.

### 4.3. Multiplex Immunofluorescence (mIF)

Here, five-micron FFPE tissue sections were cut from the 87 core biopsies and the 4 TMA blocks were then placed on standard microscope slides with a thickness of 1 mm. The fluorescent light passes through both the slide and the tissue mounted on it, and the light path is optimized for this setup. These sections underwent deparaffinization and were then subjected to heat-induced epitope retrieval in Tris-EDTA buffer (pH 9.0). A 6-plex panel multiplex immunofluorescence (mIF) assay was conducted using the following antibodies: anti-CD20 (clone L26, dilution 1:200, from Leica, Wetzlar Germany), anti-CD4 (clone EPR6855, dilution 1:200, from Abcam, Cambridge, UK), anti-CD8 (clone 4B11, dilution 1:400, from Leica), anti-FoxP3 (clone 236A/E7, dilution 1:400, from Abcam), anti-CD68 (clone 514H12, dilution 1:400, from Leica), and anti-CK (clone AE1/AE3, dilution 1:100, from Leica). Details of the antibodies and dilutions used are provided in Table 4.

Tyramide signal amplification (TSA)-conjugated fluorophores from PerkinElmer (Waltham, MA, USA) were utilized to visualize each biomarker: Opal 480 (for CD20), Opal 520 (for CD4), Opal 570 (for CD8), Opal 620 (for FOXP3), Opal 690 (for CD68), and Opal 780 (for CK AE1/AE3). Additionally, DAPI was employed as a counterstain. These biomarkers are fluorescent, and were in an FFPE section with antibodies raised against them, followed by HRP-linked secondary antibodies, and finally fluorophores. Whole slides were scanned at 0.5 um/pixel (20×) magnification using a Vectra Polaris Automated Quantitative Pathology Imaging systems. Stained multiplex slides were exposed at each of the seven filters (DAPI, Cy5, FITC, Cy3, Texas Red, Opal 570 and Opal 690), ensuring that each slide remained in focus throughout the scanning process. Nominal focus was set using a live-view on a representative slide. The scanner then screened the slide for intensity changes and created a binary map indicating where tissue was present. The scanner captured images where tissue was detected using that map. Autofocus started at the preset nominal focus and steps either side, choosing the brightest intensity level as the optimal focus. Images were reviewed and rescanned if required. The imaging speed was 12 min per square centimeter of tissue, which equates to processing around 5 slides per hour. Table 5 shows the seven filters used and the duration of exposure to each of them.

### 4.4. Selecting Regions of Interest (ROIs) Using the Phenochart Software

**Figure 5:**
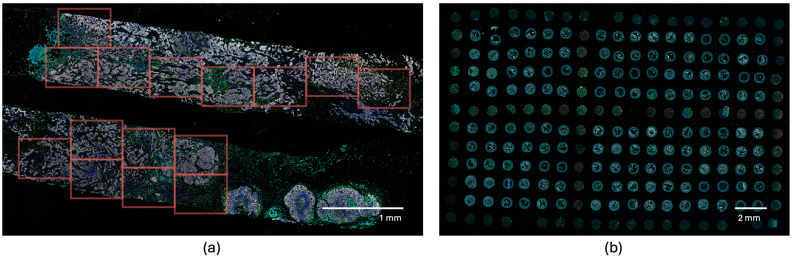
Selecting regions of interest (ROIs) using the Phenochart software (version 1.0.11) in a core (**a**) and TMA section (**b**).

Scanned fluorescent digital slides were examined and annotated manually using Phenochart software (version 1.0.11), a whole slide contextual viewer from Akoya Biosciences, Inc. (Marlborough, MA, USA). In this process, regions of interest (ROIs) were carefully selected by pathologists, focusing on areas within the tumor microenvironment while avoiding necrotic and non-invasive regions. For the tissue microarray (TMA), a TMA grid was superimposed onto the whole slide. This grid featured an adjustable number of rows and columns to match the TMA template. Each core within the grid was assigned a specific identifier composed of a number and a letter (e.g., 4F). This grid arrangement was consistently applied to each duplicate slide, ensuring consistency and accuracy in core identification across all slides, Figure 5.

### 4.5. Image Analysis Using InForm Software

**Figure 6:**
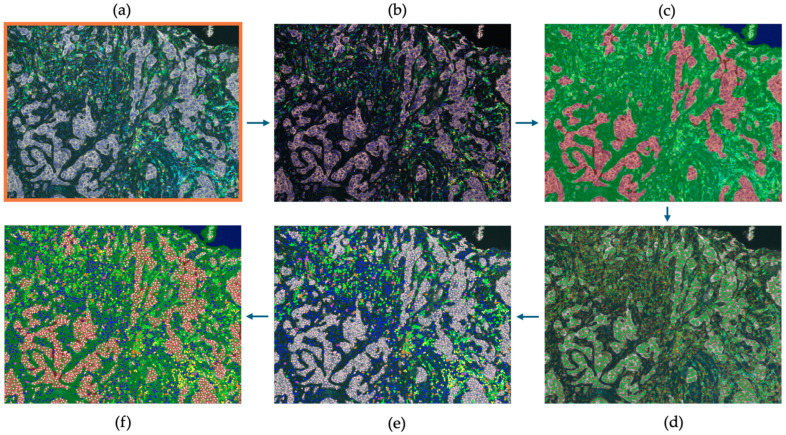
Image analysis of cores using inForm software (version 2.4.3): (**a**) selecting ROIs; (**b**) image preparation; (**c**) tissue segmentation; (**d**) cell segmentation; (**e**) phenotyping; (**f**) showing a composite view the previous steps. ROIs measured 0.66 × 0.5 mm.

**Figure 7:**
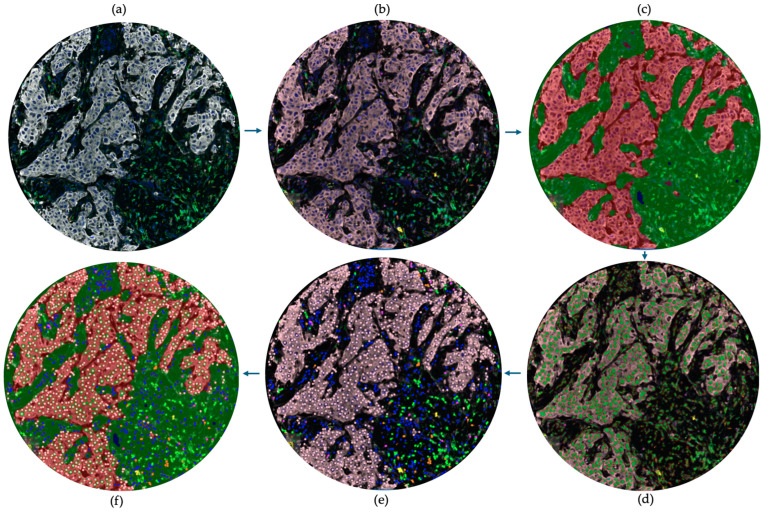
Image analysis of TMA using inForm software (version 2.4.3): (**a**) selecting ROIs; (**b**) image preparation; (**c**) tissue segmentation; (**d**) cell segmentation; (**e**) phenotyping; (**f**) showing a composite view the previous steps. All cores’ diameters were 0.6 mm.

The selected regions of interest (ROIs) were analyzed using InForm (version 2.4.3), a trainable machine learning based image analysis software (Akoya Biosciences, Inc., Marlborough, MA, USA), through five stages: image preparation, tissue segmentation, cell segmentation, phenotyping, and data export, as illustrated in Figure 6 and Figure 7. Image Preparation: Images underwent an analysis pipeline that included correction for autofluorescence and spectral library deconvolution to enhance accuracy. Tissue Segmentation: Tissue was segmented into tumor, stroma, and background regions. A machine learning algorithm was trained by making representative annotations for each tissue category. The algorithm was then applied to the remaining images. Cell Segmentation: Cell segmentation primarily relied on identifying nuclei stained with DAPI. Other markers (primarily membranous, except for FOXP3, which is nuclear) were used as supportive factors in segmenting the nuclei.

Phenotyping: A threshold-based machine learning classifier was trained with the manual annotation of cells for each phenotype. Each cell type was assigned a specific color: CD4+ cells (green), CD68+ cells (magenta), CD8+ cells (orange), FOXP3+ cells (cyan), CD20+ cells (yellow), CK+ cells (pink), and any other nuclei (dark blue, DAPI stained). Data Export: Each case’s ROIs were processed with the specific trained protocol, and all data were exported. The cell-level output data included tissue category (tumoral/stromal), phenotype expression data and x–y co-ordinates for each cell. Additionally, 13 views were generated for each ROI, including immunofluorescent, simulated brightfield, tissue segmentation map, cell segmentation map, phenotyping map, composite map, and immunostaining view for each of the 7 stains.

### 4.6. Data Analysis Using Phenoptrreports Package in R Studio

Data exported from InForm software (from ROIs of each case) were subjected to further analysis in R using the phenoptrReports package (Akoya Biosciences, Inc.). Data were merged, consolidated, and analyzed. The latter involved defining the immune cells to be studied based on the staining pattern set by the pathologists (Table 2). The generated sheets contained the following metrics: number of fields (ROIs), cell counts, cell percentages, and cell density per mm^2^. These metrics were categorized into stromal, tumoral, and total; the latter refers to the sum of stromal and tumoral data. Counts alone were used to compute percentages for each immune cell phenotype, while counts in conjunction with surface area were utilized to calculate the density of each immune cell phenotype. The percentage of each type of immune cell was calculated based on the total count of cells, including those not belonging to any of the immunostained populations. In every sample, the cell phenotype with the highest percentage was designated as the dominant phenotype, while the second highest was designated as the second predominant phenotype within that sample.

### 4.7. Defining the Immune Cell Phenotypes

The tumor microenvironments of 230 samples underwent characterization using multiplex immunofluorescence, employing a panel of 6 markers: CD4, CD8, CD20, CD68, FOXP3 and CK. Based on the observed staining patterns, immune cells were classified into distinct categories: T-helper cells (Th)—CD4+ and FOXP3-, T-regulatory cells (Treg)—CD4+ and FOXP3+, T-cytotoxic cells (Tc)—CD8+, B-cells—CD20+, Tumor-associated macrophages (TAM)—CD68+, and FOXP3—FOXP3+ and negative to the other markers. In addition to these 6 populations, another 2 categories were calculated to include in the analysis; IMM, referring to the sum of these 6 phenotypes, and ALL, which represents IMM in addition to any other cell present in the tumor microenvironment (Table 6).

### 4.8. Statistical Analysis

Statistical analysis was carried out using IBM SPSS (version 29) software (IBM Corp, Armonk, NY, USA). Descriptive statistics were performed for patient demographics and clinical characteristics. For continuous variables, median, range values and quartiles were computed. The Mann–Whitney and Kruskal–Wallis nonparametric tests were used to study the distributions of continuous variables across groups defined by clinicopathologic characteristics. The Spearman’s rank correlation coefficient was used to study the correlation between continuous variables, including overall survival (OS). OS was defined as the time from first diagnosis to the date of last being seen. Alive patients were censored at the date of last follow-up. The Kaplan–Meier survival curve was used to compare survival among different groups. All reported *p* values are two-sided, and the significance level was set at *p* < 0.05.

## 5. Conclusions

In conclusion, our study sheds light on the diverse landscape of immune cell populations within the breast cancer microenvironment, emphasizing the importance of considering ethnic diversity. We show that small tumor samples (such as core biopsies and TMA cores) are optimal for the comprehensive profiling of the immune microenvironment. Through detailed profiling across various regions, we gained valuable insight into the dynamic interplay between immune cells, histopathological parameters and tumor progression. By understanding the intricate mechanisms underlying immune cell interactions and their impact on clinical outcomes, our research paves the way for the development of personalized immunotherapeutic approaches tailored to the unique needs of breast cancer patients.

## Figures and Tables

**Figure 2:**
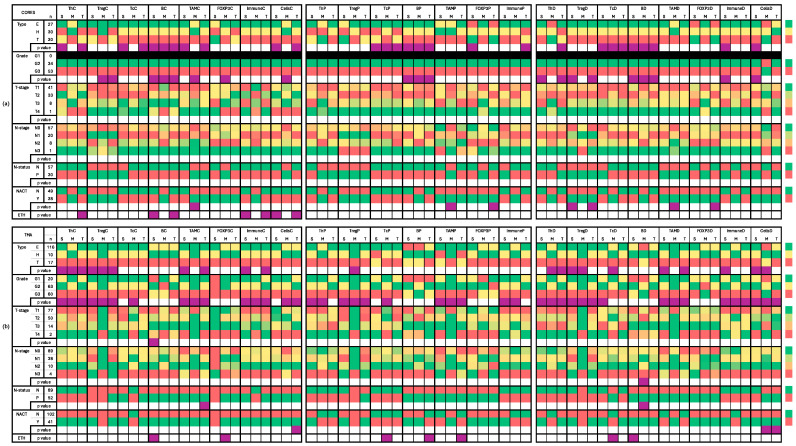
Heat map presenting the distribution of the immune cells in relation to pathologic characteristics in core samples (**a**) and TMA samples (**b**). [C] count; [P] percentage; [D] density; [S] stromal region; [M] tumoral region; [T] total ‘sum of stromal and tumoral’; Type [E: Luminal], [H: HER2 positive] and [T: TNBC]; [ETH] ethnicity. Color code reference is presented in the column to the far right of the figure. Median values are presented in colored boxes, where **RED** is the highest value and **GREEN** is the lowest value among different categories of each variable. **PURPLE** color refers to statistically significant (*p* < 0.05) different distributions among the categories of each variable.

**Figure 3:**
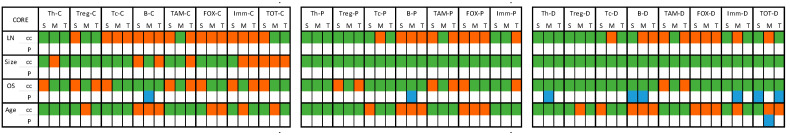
Heat map presenting the correlation of the immune cells with pathologic characteristics in core samples. [C] count; [P] percentage; [D] density; [S] stromal region; [M] tumoral region; [T] total ‘sum of stromal and tumoral’; [cc] correlation coefficient; [*p*] *p* value. Color code: **GREEN** indicates a negative correlation coefficient value with number of involved LNs and tumor size, while positive correlation with OS and age at diagnosis. **ORANGE** indicates the opposite to green correlations. **BLUE** highlights statistically significant (*p* < 0.05) correlations.

**Figure 4:**
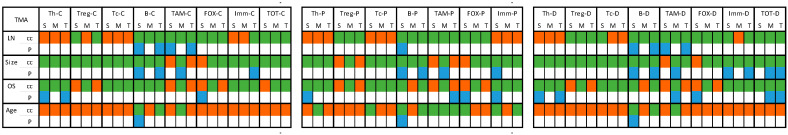
Heat map presenting correlation of the immune cells to pathologic characteristics in TMA samples. [C] count; [P] percentage; [D] density; [S] stromal region; [M] tumoral region; [T] total ‘sum of stromal and tumoral’; [cc] correlation coefficient; [*p*] *p* value. Color code: **GREEN** indicates a negative correlation coefficient value with the number of involved LNs and tumor size, while a positive correlation with OS and age at diagnosis. **ORANGE** indicates the opposite to green correlations. **BLUE** highlights statistically significant (*p* < 0.05) correlations.

**Table 1:** Patient demographics and histopathological characteristics of the studied cohort.

		Core Samples *n* = 87		TMA Samples *n* = 143		Patient Cohort *n* = 192	
		N	%	N	%	N	%
Age at Diagnosis ‘Median (IQR)’				52 (44:59)			
Molecular Type							
	Luminal	27	31	116	81.1	124	64.6
	Her2-positive	30	34.5	10	7	34	17.7
	TNBC	30	34.5	17	11.9	34	17.7
Tumor Grade							
	Grade 1	0	0	20	14	20	10.4
	Grade 2	34	39	63	44	82	42.7
	Grade 3	53	61	60	42	90	46.9
T. Size ‘Median (IQR)’		21 (15:28)		19 (12:30)		19 (14:28)	
	T-Stage						
	T1	41	47.7	77	53.8	104	55.3
	T2	33	38.4	50	35	64	34
	T3	8	9.3	14	9.8	18	9.6
	T4	1	1.2	2	1.4	2	1.1
	N/A	1				4	
Lymph nodes							
	Nodal Status						
	Positive	30	34.5	52	36.4	71	37
	Negative	57	65.5	91	63.6	121	63
	N-Stage						
	N0	57	66.3	91	63.6	121	63.4
	N1	20	23.2	38	26.6	52	27.2
	N2	8	9.3	10	7	13	6.8
	N3	1	1.2	4	2.8	5	2.6
	N/A	1				1	
Chemotherapy							
	Received	38	43.7	41	28.7	66	34.4
	Not Received	49	56.3	102	71.3	126	56.6
NPI * ‘Median (IQR)’		4.47 (3.52:5.16)		4.3 (3.36:4.9)		4.3 (3.37:4.92)	
OS	‘Median (IQR)’	105 (78:145)		101 (84:117)		103 (86.25:124:75)	

* Nottingham Prognostic Index.

**Table 2:** Distribution of cohort’s ethnic origin, and its 5-year and 10-year OS.

Ethnicity	Number	Ethnic Group	5YS *	10YS *
Pakistani	66 (34.4%)	Asian *n* = 128 (66.6%)	90.5%	81%
Indian	42 (21.9%)			
Chinese	6 (3.1%)			
Bangladeshi	1 (0.5%)			
Asian (other)	13 (6.8%)			
Caribbean	24 (12.5%)	Black *n* = 46 (24%)	85%	71%
African	14 (7.3%)			
Black (other)	8 (4.2%)			
Mixed	8 (4.2%)	OTHER *n* = 18 (9.4%)	83.5%	73%
Any other	10 (5.2%)			

* 5-year OS; 10-year OS.

**Table 3:** Dominant phenotypes in the studied samples.

Immune Phenotype		Cores		TMA	
		N	%	N	%
Th	Dominant	62	71.3	99	69.2
	2nd most predominant	17	19.5	27	18.9
Treg	Dominant	0	0	0	0
	2nd most predominant	1	1.1	0	0
Tc	Dominant	13	14.9	9	6.3
	2nd most predominant	40	46	87	60.8
B	Dominant	5	5.7	27	18.9
	2nd most predominant	11	12.6	15	10.5
TAM	Dominant	6	6.9	8	5.6
	2nd most predominant	18	20.7	14	9.8
FOXP3	Dominant	1	1.1	0	0
	2nd most predominant	0	0	0	0

**Table 4:** Details of used antibodies.

Marker	Company	Clone	Host	Marker Dilution	Reporter	Reporter Dilution	Antigen Retrieval Method and Time (min)
CD20	Leica	L26	Mouse	1:200	Opal 480	1:150	H2 (20)
CD8	Leica	4B11	Mouse	1:400	Opal 570	1:100	H1 (20)
CD4	Abcam	EPR6855	Rabbit	1:200	Opal 520	1:150	H1 (20)
FOXP3	Abcam	236A/E7	Mouse	1:400	Opal 620	1:150	H1 (20)
CD68	Leica	514H12	Mouse	1:400	Opal 690	1:150	H1 (20)
CK	Leica	AE1/AE3	Mouse	1:100	Opal 780	1:10	H1 (20)

**Table 5:** Used filters and durations of exposure.

Filter	Duration (ms)
DAPI	1.31
Cy5	8.26
FITC	3.06
Cy3	16.95
Texas Red	61.25
Opal 570	22.65
Opal 690	12.78

**Table 6:** Defining cell phenotypes in phenoptrReports according to staining pattern.

Cell Phenotype	Defined Staining Pattern
T-helper cells (Th)	CD4+/FOXP3-
T-regulatory cells (Treg)	CD4+/FOXP3+
T-cytotoxic cells (Tc)	CD8+/FOXP3±
B-cells	CD20+/FOXP3±
Tumor-associated macrophages (TAMs)	CD68+/FOXP3±
FOXP3-positive cells	FOXP3+/CD4-/CD8-/CD20-/CD68-
IMM cells	Sum of Th, Treg, Tc, B, TAMs and FOXP3
ALL cells	Sum of IMM and DAPI+ cells (other TME cells)

## Data Availability

**Statement:** All study data are held internally by the host institution and are available upon specific request to the authors with agreement from the host institution.

## Supplementary Materials

The following supporting information can be downloaded at: https://www.mdpi.com/article/10.3390/ijms25126501/s1.
