# Supplementary material for: Detailed Profiling of the Tumor Microenvironment in Ethnic Breast Cancer, Using Tissue Microarrays and Multiplex Immunofluorescence
Source: Int J Mol Sci. 2024 Jun 13;25(12):6501. doi: 10.3390/ijms25126501 (PMC11203983; doi:10.3390/ijms25126501)
Supplement: Supplementary file 1 [file ijms-25-06501-s001.zip › Supplementary Figure S2.pdf]

Cell	Core samples	TMA samples
Th		
Tc		
B		

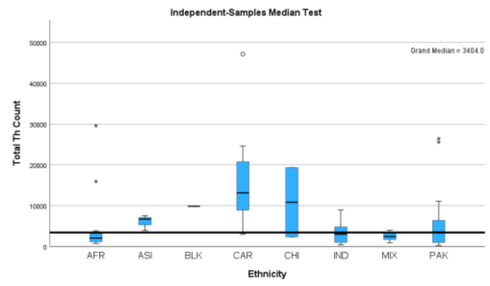

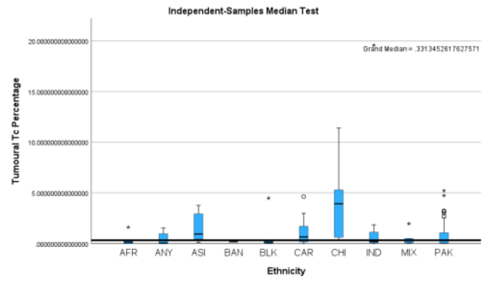

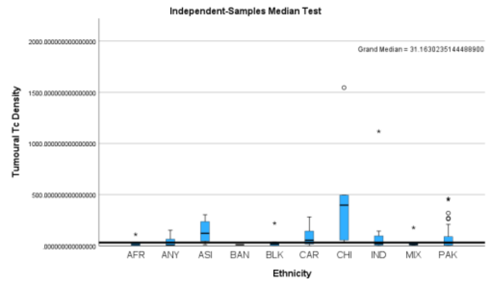

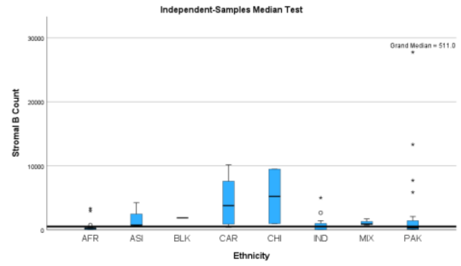

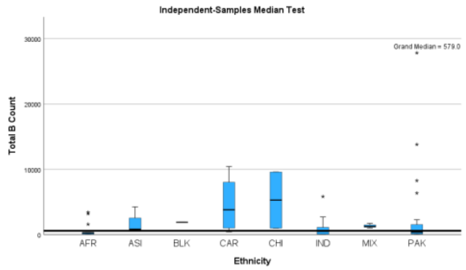

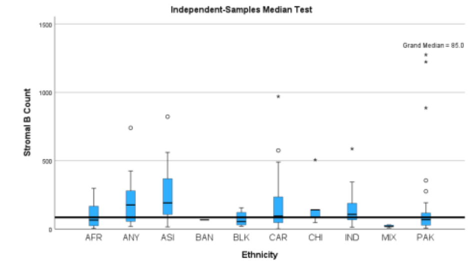

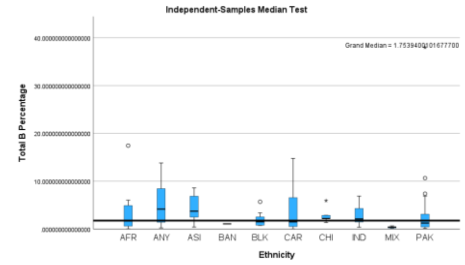

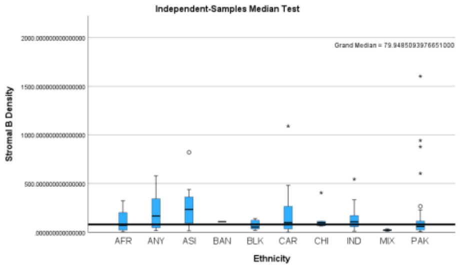
Independent-Samples Median Test
Grand Median = 79.9485093976651000
Stromal B Density
Ethnicity
AFR ANY ASI BAN BLK CAR CHI IND MIX PAK

TAM*

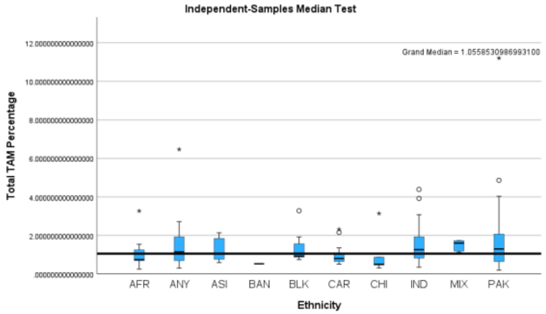
Independent-Samples Median Test
Grand Median = 1.0558530986993100
Total TAM Percentage
Ethnicity
AFR ANY ASI BAN BLK CAR CHI IND MIX PAK

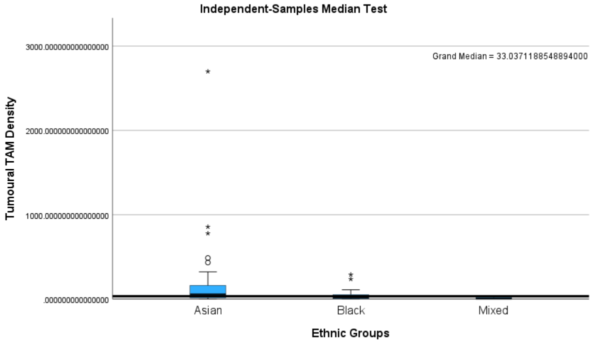
Independent-Samples Median Test
Grand Median = 33.0371188548894000
Tumoural TAM Density
Ethnic Groups
Asian
Black
Mixed

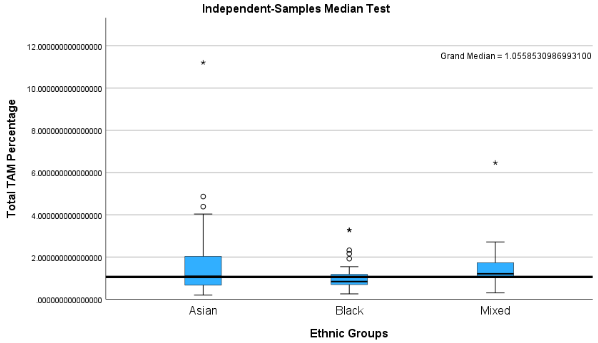
Independent-Samples Median Test
Grand Median = 1.0558530986993100
Total TAM Percentage
Ethnic Groups
Asian
Black
Mixed

FOXP3

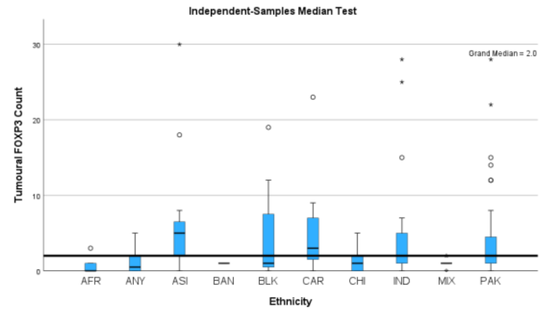
Independent-Samples Median Test
Grand Median = 2.0
Tumoural FOXP3 Count
Ethnicity
AFR ANY ASI BAN BLK CAR CHI IND MIX PAK

IMM

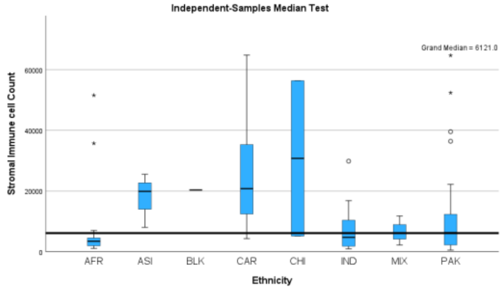

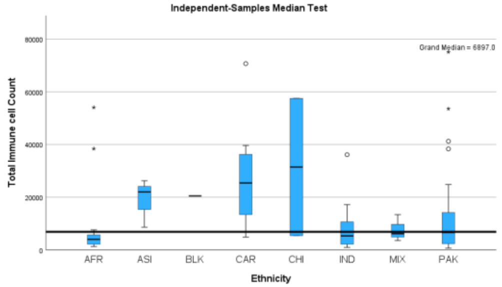

ALL

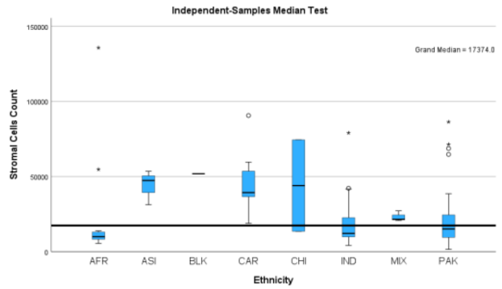

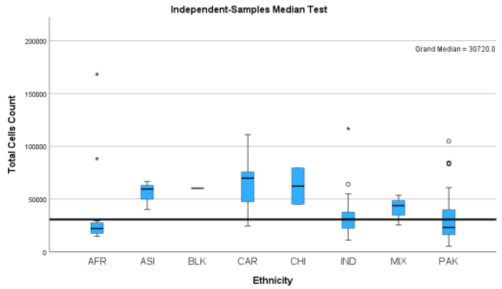

Figure S2. Box-Plots featuring significant differences (with corresponding p values) of immune cells distribution among different ethnicities, except TAMs, which in addition shows the only significant differences among the three Ethnic groupings (Asian, Black and OTHER)
